# In Vitro Assessment of ^177^Lu-Labeled Trastuzumab-Targeted Mesoporous Carbon@Silica Nanostructure for the Treatment of HER2-Positive Breast Cancer

**DOI:** 10.3390/ph17060732

**Published:** 2024-06-05

**Authors:** Ayça Tunçel, Simone Maschauer, Olaf Prante, Fatma Yurt

**Affiliations:** 1 Department of Nuclear Applications, Institute of Nuclear Science, Ege University, Bornova 35100, Turkey; nuk.ayca.tuncel@gmail.com; 2 Department of Nuclear Medicine, Molecular Imaging and Radiochemistry, Friedrich-Alexander University Erlangen-Nürnberg (FAU), Ulmenweg 18, D-91054 Erlangen, Germany; simone.maschauer@uk-erlangen.de

**Keywords:** ^177^Lu, mesoporous carbon and silica nanomaterials, HER2+ breast cancer, radiolabeling

## Abstract

This study assessed the effectiveness of a trastuzumab-targeted ^177^Lu-labeled mesoporous Carbon@Silica nanostructure (DOTA@TRA/MC@Si) for HER2-positive breast cancer treatment, focusing on its uptake, internalization, and efflux in breast cancer cells. The synthesized PEI-MC@Si nanocomposite was reacted with DOTA-NHS-ester, confirmed by the Arsenazo(III) assay. Following this, TRA was conjugated to the DOTA@PEI-MC@Si for targeting. DOTA@PEI-MC@Si and DOTA@TRA/MC@Si nanocomposites were labeled with ^177^Lu, and their efficacy was evaluated through in vitro radiolabeling experiments. According to the results, the DOTA@TRA/MC@Si nanocomposite was successfully labeled with ^177^Lu, yielding a radiochemical yield of 93.0 ± 2.4%. In vitro studies revealed a higher uptake of the [^177^Lu]Lu-DOTA@TRA/MC@Si nanocomposite in HER2-positive SK-BR-3 cells (44.0 ± 4.6% after 24 h) compared to MDA-MB-231 cells (21.0 ± 2.3%). The IC_50_ values for TRA-dependent uptake in the SK-BR-3 and BT-474 cells were 0.9 µM and 1.3 µM, respectively, indicating affinity toward HER-2 receptor-expressing cells. The lipophilic distribution coefficients of the radiolabeled nanocomposites were determined to be 1.7 ± 0.3 for [^177^Lu]Lu-DOTA@TRA/MC@Si and 1.5 ± 0.2 for [^177^Lu]Lu-DOTA@PEI-MC@Si, suggesting sufficient passive transport through the cell membrane and increased accumulation in target tissues. The [^177^Lu]Lu-DOTA@TRA/MC@Si nanocomposite showed an uptake into HER2-positive cell lines, marking a valuable step toward the development of a nanoparticle-based therapeutic agent for an improved treatment strategy for HER2-positive breast cancer.

## 1. Introduction

Breast cancer is a significant global health issue, with the highest number of cancer-related deaths among women [1]. Breast tumors characterized by the overexpression of the human epidermal growth factor receptor 2 (HER2) protein are associated with aggressive cancers and poor clinical outcomes [2]. HER2 plays a crucial role in predicting and prognosing conditions, especially in breast and gastric cancers. Due to its varied expression in different tumors, accurately determining HER2 status is of essential importance [3]. Monoclonal antibodies against HER2 such as trastuzumab have been used to enhance the specificity of radiotherapy with nanoparticles [4].

Targeted radiotherapy with radiolabeled nanoparticles has emerged as a promising approach for the treatment of HER2-positive breast cancer as it provides a targeted delivery method that reduces damage to healthy tissue [5,6,7]. Studies have explored the use of HER2-targeted nanoparticles for the delivery of therapeutic agents such as paclitaxel and doxorubicin to HER2-positive breast cancer cells [8,9]. Several preclinical studies have shown that radiolabeled trastuzumab, using various radionuclides like zirconium-89, copper-64, iodine-131, lutetium-177, and indium-111, achieves high receptor saturation and tumor uptake [10,11] For instance, in a biodistribution study, ^89^Zr-trastuzumab exhibited strong HER2-specific tumor uptake and even detected previously undetected brain metastases [12,13]. Other research has demonstrated the potential of imaging HER2 with ^89^Zr-trastuzumab for assessing HER2 status, understanding tumor heterogeneity, and predicting responses to anti-HER2 treatments [14,15]. A phase I study of ^177^Lu-trastuzumab in patients with HER2-positive primary and metastatic breast lesions has also reported a similar specific uptake [16].

Lutetium-177 (^177^Lu), with its maximum beta energy of 0.5 MeV and a relatively short half-life of 6.7 days, is routinely used in nuclear medicine as a therapeutic isotope. ^177^Lu is also a radionuclide that can be used for both therapeutic and imaging purposes. It emits beta particles, which are useful for targeted radiotherapy, and gamma rays, which can be used for imaging [17,18]. For the labeling of biomolecules with ^177^Lu, the use of bifunctional chelating agents (BFCs) is commonly applied [17], allowing covalent binding to bio-molecules through reactive functional groups, while the chelator is available to form highly stable complexes with radiometal ions [19]. The most prominent chelator of ^177^Lu^3+^, 1,4,7,10-tetraazacyclododecane-1,4,7,10-tetraacetic acid (DOTA), can be introduced to amine-bearing biomolecules or nanoparticles by the covalent conjugation of its N-hydroxy succinimide (NHS) ester, so the DOTA-NHS ester represents a highly suitable BFC for the synthesis and design of new radiotherapeutics in nuclear medicine [20]. In addition, DOTA–trastuzumab conjugates have been effectively used in clinical settings and are widely studied. Recent studies have shown that [^177^Lu]Lu-labeled DOTA–trastuzumab conjugates exhibit high specificity for HER2-positive breast cancer treatment and demonstrate specific tumor uptake [21,22]. The study by Guleria et al. (2021) reported that [^177^Lu]Lu-DOTA-trastuzumab could be successfully used for treating HER2-positive metasta-tic breast cancer, showing high binding affinity. These studies indicate that [^177^Lu]Lu-DOTA-trastuzumab is a reliable and effective therapeutic option in clinical practice.

However, using nanomaterials in conjunction with DOTA–trastuzumab conjugates offers several significant advantages to further enhance therapeutic efficacy. Nanomaterials possess a higher surface area, allowing for a greater loading of therapeutic agents and radionuclides, leading to higher local concentrations at the tumor site. Additionally, nano-ma-te-ri-als can be engineered to improve their stability, biocompatibility, and circulation time, thus enhancing their ability to target and penetrate tumors more effectively [23,24,25,26,27]. These properties contribute to a more sustained and controlled release of the therapeutic payload, reducing systemic toxicity, and improving overall treatment outcomes. Therefore, the combination of clinically proven DOTA–trastuzumab with nanomaterials provides substantial benefits for the targeted treatment of HER2-positive breast cancer cells. Therefore, the use of ^177^Lu-labeled nanoparticles targeted to HER2 has the potential to achieve high levels of specific tumor uptake, resulting in improved in vitro and in vivo efficacy compared to conventional therapies such as chemotherapy and radiation therapy [28,29,30,31,32,33,34,35,36].

Mesoporous silica nanoparticles (MSNPs) and mesoporous carbon nanoparticles (MCNPs) have unique properties such as high surface area, tunable pore size, and good biocompatibility that make them attractive for use as nanocarriers for therapeutic and diagnostic agents [23,24,25,26,27]. These nanoparticles can also be labeled with ^177^Lu using BFCs to provide a targeted approach for the delivery of radioactive doses to cancer cells [37]. There are numerous examples in the literature regarding nanoparticles labeled with Lu-177 by using BFCs. For example, in studies conducted by Kovacs et al. (2015), the DOTA-NHS-ester was conjugated to polyamidoamine (PAMAM) dendrimers. Subsequently, ^177^LuCl_3_ was added and incubated at temperatures ranging from 37 to 50 °C for 20–60 min to complete radiolabeling with a high radiochemical yield (RCY) of 98% [38]. Vats et al. (2018) developed ^177^Lu-labeled cyclic Asg–Gly–Asp peptide-tagged carbon nanospheres, confirming an RCY of 80% through TLRC analysis [39]. Zhang et al. (2020) engineered alpha-melanocyte-stimulating hormone (αMSH)-surface PEG-coated silica nanoparticles, targeting the melanocortin-1 receptor (MC1-R) on melanoma cells that were radiolabeled with ^177^Lu in a RCY of greater than 95% [40]. Another study showed that ^177^Lu-labeled PAMAM dendrimers using a DOTA-like bifunctional chelator achieved a high RCY, stability, and greater than 98% radiochemical purity [41]. Ge et al. (2022) reported on the development of a general radiolabeling method for Fe_3_O_4_ nanoparticles with both diagnostic and therapeutic radiometal isotopes. These authors described diphosphonate-polyethylene glycol (DP-PEG) decorated particles that were ^177^Lu-labeled with 50% RCY and characterized the ^177^Lu-labeled Fe_3_O_4_ nanoparticles as well as their in vitro and in vivo efficacy against tumor cells [42].

Interestingly, the study by Cai et al. (2017) focused on the potential of ^177^Lu-labeled trastuzumab (TRA)-targeted AuNPs for radiotherapy [29]. The authors described the preparation and characterization of the ^177^Lu-TRA-AuNPs as well as their in vitro and in vivo efficacy against tumor cells. They found that ^177^Lu-TRA-AuNPs demonstrated effective binding and internalization in HER2-positive SK-BR-3, BT-474, and MDA-MB-361 cells. ^177^Lu-TRA-AuNPs were significantly more potent than ^177^Lu-AuNP in inhibiting tumor growth in mice, presenting a promising option for targeted radiation treatment of HER2-positive breast cancer. ^177^Lu-TRA-AuNPs exhibited high tumor uptake and good targeting ability, leading to improved therapeutic efficacy compared to non-targeted radiotherapy [29]. The results showed that the ^177^Lu-labeled nanoparticles could target and accumulate in HER2-positive tumors, and the treatment was well-tolerated with limited side effects. However, the efficacy of ^177^Lu labeled mesoporous carbon@silica nanoparticles targeted with trastuzumab for the treatment of HER2-positive breast cancer has not been adequately evaluated yet. This study aims to address this deficiency and assess the therapeutic potential of this novel combination.

In this study, we aimed to radiolabel and evaluate in vitro the HER2-targeting capabilities of ^177^Lu-labeled DOTA@TRA/MC@Si nanocomposites, focusing on their therapeutic applications for targeting HER2-positive breast cancer cells. Herein, we report on the in vitro investigations of the HER2-targeting capabilities of the [^177^Lu]Lu-DOTA@TRA/MC@Si nanocomposite.

## 2. Results and Discussion

### 2.1. Characterization of Synthesized Polyethyleneimine Integrated PEI-MC@Si Nanocomposite

The nanocomposite material has been successfully synthesized, with necessary characterizations performed at each stage of synthesis. The results of these characterizations have been detailed in our previous paper [43]. Briefly, dynamic light scattering (DLS), scanning electron microscopy (SEM), and transmission electron microscopy (TEM) were employed to determine the sizes of the nanomaterials synthesized at each step. The DLS analyses revealed that the average diameters of MCNp and the MC@Si nanocomposite were 90.4 ± 5.8 nm and 110.8 ± 3.9 nm, respectively. These findings were corroborated by SEM and TEM analyses, which provided further insights into the orientation of the pore structures and the morphologies of the nanomaterials on the surfaces of MCNp, MC@Si, and PEI-MC@Si. After the modification with PEI, the results indicated that the nanocomposites maintained a homogeneous distribution and spherical shape. Fourier transform infrared spectroscopy (FTIR) and UV–VIS spectrometry analyses were also conducted. These analyses revealed characteristic bands and absorption values that agreed with the literature [44]. Thermogravimetric analyses (TGA) indicated that at 600 °C, the PEI content in the PEI-MC@Si nanocomposite experienced a weight loss of approximately 39.08%. In conclusion, these characterization steps verified the successful synthesis of the nanomaterial.

### 2.2. Analysis of Primary Amine Groups on PEI-MC@Si Nanocomposites Using the Ninhydrin Method

**Figure 1:**
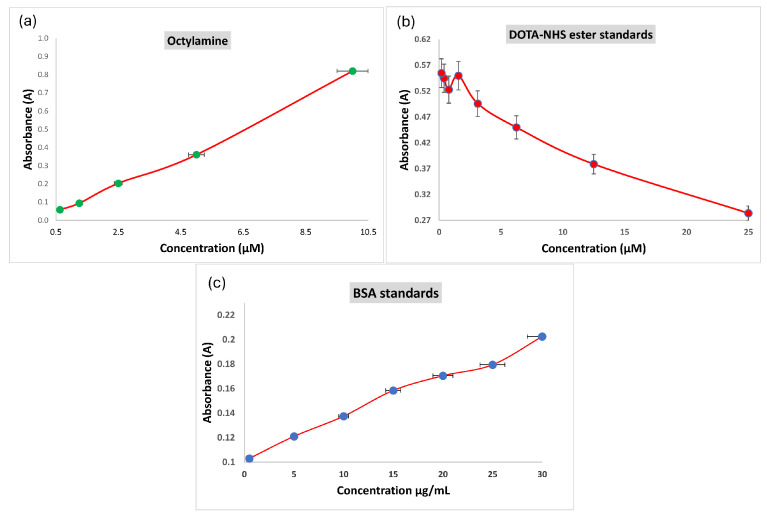
(**a**) Absorbance/concentration curve of octylamine drawn with the ninhydrin test. The green dots show different concentrations of Octylamine. (**b**) Standard calibration graph dependent on the absorption detected at 595 nm for the DOTA-NHS-ester concentration. The red dots indicate various concentrations of DOTA-NHS ester standards. (**c**) BSA standard absorbance/concentration graph. The blue dots represent different concentrations of BSA standards. Data are presented as mean values ± SD, determined in triplicate from two independent experiments.

The ninhydrin method was utilized to detect amine group concentrations on the nanocomposite, as documented by Sun et al. (2019) [45]. Tests were conducted with ninhydrin in a 60% ethanol/water mixture at various concentrations, leading to noticeable color changes. A microplate reader analyzed reactions at a 570 nm wavelength. Initial tests with octylamine, a primary amine standard, were conducted to ascertain the yield of the colored product. A concentration calibration curve was established with octylamine, and absorbance values were calculated using the derived equation from the octylamine concentration curve graph based on the ninhydrin test (Figure 1a). The amine group concentrations on the surfaces of the PEI-MC@Si and TRA/PEI-MC@Si nanocomposites were determined to be 8.0 and 2.5 nmol/mg, respectively.

### 2.3. Determination of DOTA Content of the Nanocomposites Using the Arsenazo(III) Method

Determination of the DOTA content was conducted by Sun et al. (2019) [46]. The Cu: Arsenazo(III) assay provides a straightforward technique for determining the concentration of DOTA (Figure 1b). Supernatant samples of the DOTA@PEI-MC@Si nanocomposite, which were prepared under identical conditions after synthesis, were tested. The wells exhibited a bright blue color, distinct from the deep blue observed in the Cu: Arsenazo calibration graph. This color change indicated the presence of excess unreacted DOTA-NHS-ester chelator in the supernatants Absorbance data showed minimal concentrations of the DOTA-NHS-ester in the supernatant sample of the DOTA@PEI-MC@Si nanocomposite, with 90.2 ± 2.8% of the initially added DOTA-NHS-ester successfully reacting with the free amine functional groups on the nanocomposite structure. In contrast, the supernatant sample of the DOTA@PEI-MC@Si nanocomposite had DOTA-NHS-ester concentrations exceeding 225 nM, with only 10.3 ± 3.5% of it reacting with the free amine functional groups.

### 2.4. Trastuzumab Conjugation to DOTA@PEI-MC@Si Nanocomposite

The BCA method was performed to detect protein concentrations in the supernatant obtained after trastuzumab was modified to react with the DOTA@PEI-MC@Si nanocomposite (DOTA@TRA/MC@Si). Absorption values of the BSA standards were used to draw a standard concentration graph from which an equation was derived (Figure 1c). The amount of TRA in the supernatant of the DOTA@TRA/MC@Si nanocomposite was calculated to be 86.1 µg/mL, with the initial amount of trastuzumab added to the DOTA@PEI-MC@Si nanocomposite being 125.1 µg/µL. Consequently, the trastuzumab binding yield to the DOTA@PEI-MC@Si nanocomposite was calculated as 31.1 ± 4.5%.

### 2.5. Radiolabeling of the Nanocomposite with ^177^Lu and Quality Control Results

**Figure 2:**
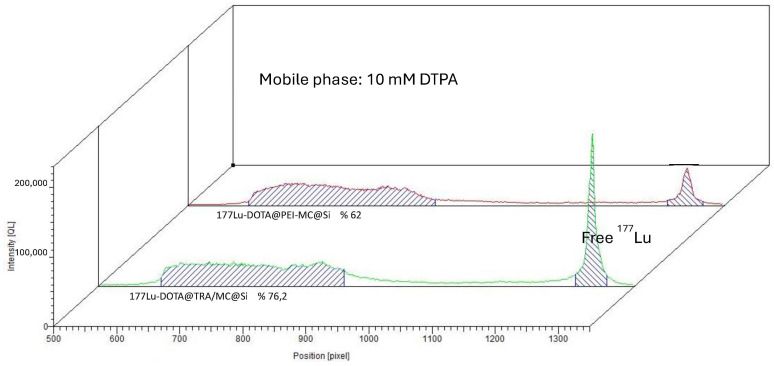
ITLC chromatograms obtained for [^177^Lu]Lu-DOTA@TRA/MC@Si in a 10 mM DTPA chamber solution.

**Figure 3:**
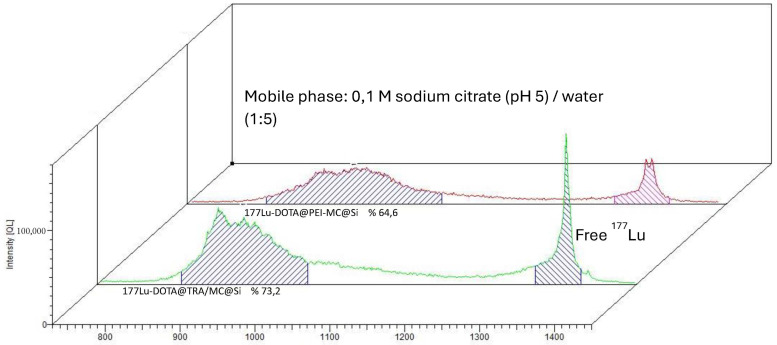
ITLC chromatograms obtained for [^177^Lu]Lu-DOTA@TRA/MC@Si in a 0.1 M sodium citrate (pH 5)/water (1:5) chamber solution.

**Figure 4:**
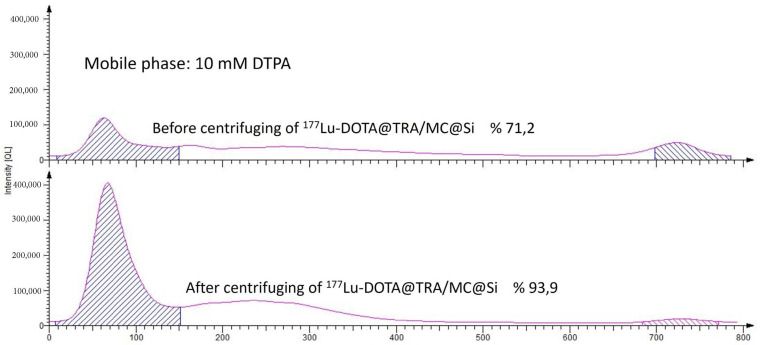
ITLC chromatograms obtained for [^177^Lu]Lu-DOTA@TRA/MC@Si after centrifugation in a 10 mM DTPA chamber solution.

**Figure 5:**
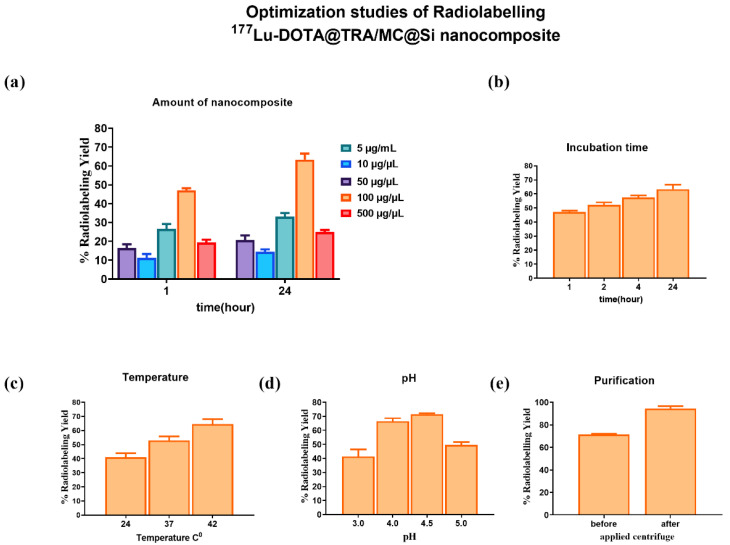
Evaluation of reaction parameters for the radiolabeling of DOTA@TRA/MC@Si nanocomposite with ^177^Lu and determination of the RCY of the [^177^Lu]Lu-DOTA@TRA/MC@Si nanocomposite: (**a**) amount of nanocomposite, (**b**) incubation time, (**c**) temperature, (**d**) pH, and (**e**) purification by ultracentrifugation. Data are presented as the mean values ± SD, determined in triplicate from four independent experiments.

The DOTA@TRA/MC@Si nanocomposite was radiolabeled with ^177^Lu, and subsequent quality control studies were conducted using the TLRC method. The results showed that in both chamber solutions, the free ^177^Lu moved along the ITLC-SG strip with an Rf value of approximately 0.9, while the [^177^Lu]Lu-DOTA@TRA/MC@Si exhibited a peak at the start with an Rf value of 0.1. Figure 2 and Figure 3 respectively present the results obtained from the 10 mM DTPA and 0.1 M sodium citrate (pH 5)/water (1:5) chamber solutions. To remove the unbound free ^177^Lu from the radiolabeled compound (which had a yield of 75%), ultrafiltration was conducted using a Falcon tube with a 100 kDa MWCO, at 3000 rpm for 15 min. This purification process resulted in a radiochemical purity of approximately 93 ± 2 (Figure 4). In the study, we also optimized the parameters influencing the RCY including the incubation time, nanocomposite amount, reaction temperature, and pH variation, which is shown in Figure 5. An initial investigation into the effect of the DOTA@TRA/MC@Si nanocomposite amount on labeling efficiency was conducted by testing different concentrations of the nanocomposite (5, 10, 50, 100, 500 µg/µL). The highest RCY, ranging from 63 to 68%, was achieved with a 100 µg/µL nanocomposite. The study further assessed the impact of different pH levels (3, 4, 4.5, 5) on the RCY, observing the highest yield of 66–71% at pH 4.5. Other examined parameters included incubation times (1, 2, 4, and 24 h) and temperatures (room temperature, 37 °C, and 42 °C). The findings revealed no increase in RCY after the first and second hour; however, longer incubation times (24 h) and a temperature increase to 42 °C during radiolabeling did result in increased yields. It is important to note that we obtained similar results when the nanoparticle, which was not targeted with trastuzumab [^177^Lu]Lu-DOTA@PEI-MC@Si, was labeled under the same conditions.

In a study conducted by Zhang et al. (2022), they addressed the radiolabeling of upconverting nanoparticles (UCNP) targeted with trastuzumab with ^177^Lu, which potentially plays a role in imaging and treating lymph node metastasis [36]. In their study, ^177^Lu-Np-mAb was formed as a result of chelating ^177^Lu to the bisphosphate groups of Np-mAb. For this purpose, 20 µL of ^177^LuCl_3_-HCl solution (18.5–37 mBq), dissolved in 20 µL of 0.25 M sodium acetate, was added to the Np-mAb solution (1 mg/mL, 0.05 mL), then the mixture was stirred at room temperature for 30 min. Then, unbound ^177^Lu was removed from these nanoparticles by centrifuging the mixture three times at 10,000 rpm for 5 min each through a 3000 K ultrafiltration tube. After the purification process, similar to this study, the radiochemical purity of ^177^Lu-Np-mAb, as measured by TLRC, was in the range of 93–95%. In a study by Salvanou et al. (2022), after modifying magnetic iron oxide nanoparticles (MION) with alginic acid and polyethylene glycol (PEG), they successfully performed radiolabeling with ^68^Ga and ^177^Lu [34], also achieving an RCY of 93.7–95.2%. When compared with the literature, the results obtained by this study were very similar.

### 2.6. Stability and Lipophilicity Results of [^177^Lu]Lu-DOTA@TRA/MC@Si Nanocomposite

The radiolabeled nanocomposite was prepared under the optimum radiolabeling conditions (42 °C, pH 4.5, 100 µg/µL nanocomposite amount, and 24-h incubation period) for the stability test. The RCY was found to be between 65 and 70% after 24 h. Thus, the radiochemical purity was increased to 93 ± 2.4% by ultracentrifugation, so that further in vitro studies on the stability of the [^177^Lu]Lu-DOTA@PEI-MC@Si nanocomposite in serum could be performed. For comparison, the [^177^Lu]Lu-DOTA@PEI-MC@Si nanocomposite (with TRA) was used. Table 1 and Table 2 present the results of the stability tests for both [^177^Lu]Lu-DOTA@TRA/MC@Si and [^177^Lu]Lu-DOTA@PEI-MC@Si in human serum. For this purpose, both radiolabeled nanocomposites were formulated in PBS (pH 7.4) and incubated in 10% HSA solutions at a temperature of 37 °C. A control experiment was conducted at room temperature in PBS (pH 7.4). The results after various incubation time intervals (0, 1, 24, 48, and 72 h) indicated that the radiochemical purity of both radiolabeled nanocomposites slightly decreased with increasing incubation time, however, the stability exceeded 75% after 24 h. In the assessment of radiochemical stability, it was observed that the nanocomposites exhibited consistent stability levels without the significant displacement of ^177^Lu^3+^ across all temperatures at the baseline time point. Upon further incubation at 37 °C in PBS, the observed radiochemical purity remained relatively high, suggesting a stable profile under physiological conditions. Conversely, a comparative analysis at room temperature revealed decreased radiochemical stability, emphasizing the significance of temperature as a determinant factor in the stability of radiolabeled nanocomposites. These results indicated a decrease in radiochemical purity over time, especially under room temperature conditions. Moreover, the nanocomposites also displayed reduced stability in the HSA solution after just 2 h.

In 2022, a study by Salvanou and colleagues detailed the stability of nanomaterials named [^177^Lu]Lu-MA and [^177^Lu]Lu-MAPEG [34]. These materials were incubated in serum at room temperature for 7 days, and the results after the first 2 h showed that the stability of [^177^Lu]Lu-MA was 79.81 ± 1.28%, and [^177^Lu]Lu-MAPEG was 77.29 ± 1.69%. In addition, the lipophilicities (logD_7.4_ distribution coefficients) of [^177^Lu]Lu-DOTA@TRA/MC@Si and [^177^Lu]Lu-DOTA@PEI-MC@Si were determined to be 1.7 ± 0.34 and 1.5 ± 0.17, respectively, indicating that these nanocomposites are moderately lipophilic. Our research findings align with these results, indicating that the stability of our nanomaterial in serum was over 75% after the first hour and maintained acceptable integrity after 24 h.

### 2.7. Cellular Uptake of [^177^Lu]Lu-DOTA@TRA/MC@Si and [^177^Lu]Lu-DOTA@PEI-MC@Si Nanocomposites

**Figure 6:**
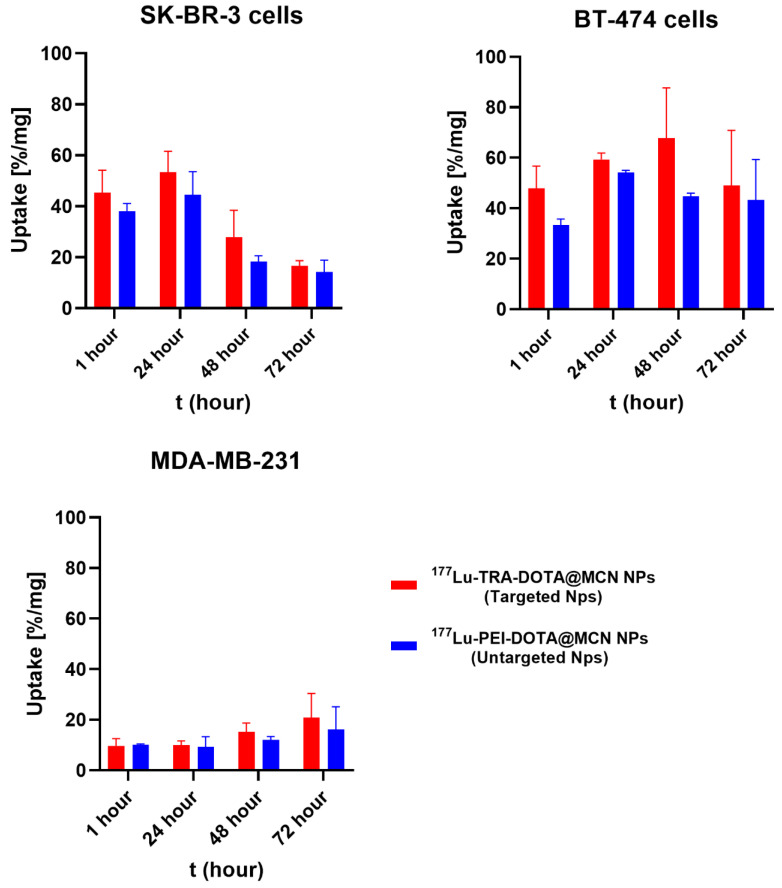
Cellular uptake of [^177^Lu]Lu-DOTA@TRA/MC@Si and [^177^Lu]Lu-DOTA@PEI-MC@Si nanocomposites in HER2-positive cells (SK-BR-3 & BT-474) and HER2-deficient cells (MDA-MB-231). Data are presented as mean values ± SD, determined in triplicate from two independent experiments.

Cellular uptake experiments were performed on the [^177^Lu]Lu-DOTA@TRA/MC@Si and [^177^Lu]Lu-DOTA@PEI-MC@Si nanocomposites across various cell lines: HER2-positive SK-BR-3, BT-474 cells, and HER2-deficient MDA-MB-231 cells. All cellular uptake values were normalized to the cell protein content as measured by the Bradford method to allow for direct comparison between the different cell lines (Figure 6).

According to the results, the uptake of [^177^Lu]Lu-DOTA@TRA/MC@Si nanocomposite in the SK-BR-3 cell line was 44 ± 4.6% in the first 24 h, decreasing to 27 ± 3.9% after 48 h and to 15 ± 1.8% after 72 h. The highest uptake in the SK-BR-3 cell line occurred at 24 h. Similarly, the highest uptake of [^177^Lu]Lu-DOTA@PEI-MC@Si nanocomposite was 38 ± 4.2% after 1 h, decreasing to 18 ± 3.6% after 48 h and to 14 ± 1.5% after 72 h. Furthermore, in the BT-474 cell line, the uptake was found to be higher for both nanocomposites at each time interval (1, 24, 48, 72 h) compared to the other cell lines. Particularly, after 48 h, unlike SK-BR-3, the uptake was higher than at 24 h. The highest uptake observed in the SK-BR-3 cell line was after 24 h. In contrast, in the BT-474 cell line, the highest uptake was approximately 63 ± 5.7% at 48 h. [^177^Lu]Lu-DOTA@TRA/MC@Si nanocomposite exhibited higher uptake at each time interval in the SK-BR-3 and BT-474 cell lines compared to its non-targeted version of the nanocomposite.

The results indicated that the SK-BR-3 cell line exhibited the highest uptake for [^177^Lu]Lu-DOTA@TRA/MC@Si within the initial 24 h. In contrast, the BT-474 cell line showed increased uptake for both nanocomposites for 48 h. Impressively, the targeted [^177^Lu]Lu-DOTA@TRA/MC@Si demonstrated superior performance over its non-targeted version in both the SK-BR-3 and BT-474 cells. Noteworthy, the HER2-negative MDA-MB-231 cell line revealed markedly lower uptake for both compounds, underscoring the role of cellular specificity in the effectiveness of these nanocomposites.

In a study by Zhang et al. (2022), trastuzumab was integrated into synthesized UCNP for imaging lymph node metastasis and labeled with ^177^Lu [36]. In vitro experiments were conducted on human breast cancer cells (SK-BR-3) and normal liver cells (HL-7702) to evaluate the cytotoxicity of ^177^Lu-Np-mAb. Uptake studies were conducted to confirm the targeting of NP-mAb nanoparticles with trastuzumab. SK-BR-3 cells with high HER-2 expression were incubated with NP, NP-mAb, and ^177^Lu-NP-mAb for 12 h, respectively. Similarly, MDA-MB 231 cells with low HER-2 receptor expression were incubated with ^177^Lu-NP-mAb for 12 h. Upon evaluation, ^177^Lu-NP-mAb exhibited higher uptake in SK-BR-3 cells compared to MDA-MB 231 cells [36], which is in good agreement with the results presented in this study.

### 2.8. Results of Competitive Binding Experiment of Radiolabeled Nanocomposite

**Figure 7:**
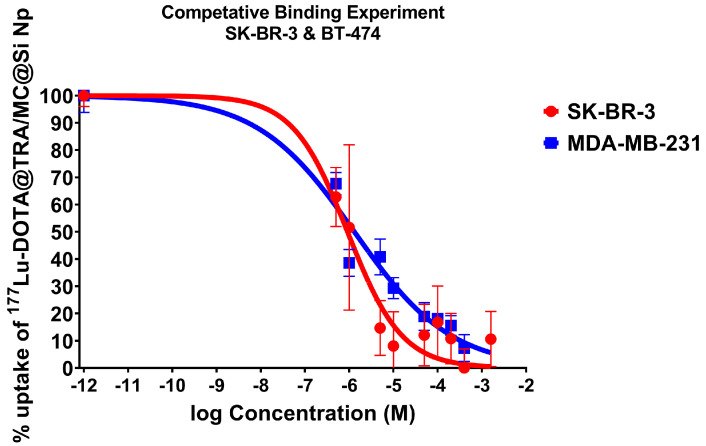
Competitive binding experiments were conducted with [^177^Lu]Lu-DOTA@TRA/MC@Si and increasing concentrations of trastuzumab using SK-BR-3 and BT-474. Data are expressed as mean values ± SD (*n* = 4) from two independent experiments.

The analysis of competitive binding experiments with [^177^Lu]Lu-DOTA@TRA/MC@Si and increasing concentrations of TRA were conducted on HER2-positive cell lines, BT-474 and SK-BR-3 (Figure 7). While for HER2-negative MDA-MB-231 cells, no competition curve was observed, competitive binding results on BT-474 and SK-BR-3 cells demonstrated the affinity of [^177^Lu]Lu-DOTA@TRA/MC@Si nanoparticles to the HER2 receptor. The results suggested that the [^177^Lu]Lu-DOTA@TRA/MC@Si nanocomposite had a high affinity to the HER2-positive SK-BR-3 and BT-474 cell lines. The IC_50_ of trastuzumab required to compete with [^177^Lu]Lu-DOTA@TRA/MC@Si were calculated as 0.9 µM (SK-BR-3 cells) and 1.3 µM (BT-474 cells), respectively. The observed high cellular uptake of [^177^Lu]Lu-DOTA@TRA/MC@Si nanoparticles in SK-BR-3 and BT-474 cells at the initial incubation phase compared to HER2-negative MDA-MB-231 cells could therefore be partly HER2-mediated and ascribed to the affinity of ^[177^Lu]Lu-DOTA@TRA/MC@Si to HER2. This result could indicate that the TRA-targeted nanoparticles demonstrated a HER2 affinity-dependent internalization capability in HER2-positive cells to some extent at 1–48 h after incubation (Figure 6). Clearly, more experiments are needed to elucidate the HER2-dependent difference in cellular uptake of TRA-targeted and non-targeted nanocomposite in BT-474 and SK-BR-3 cells.

### 2.9. Internalization and Efflux Results

**Figure 8:**
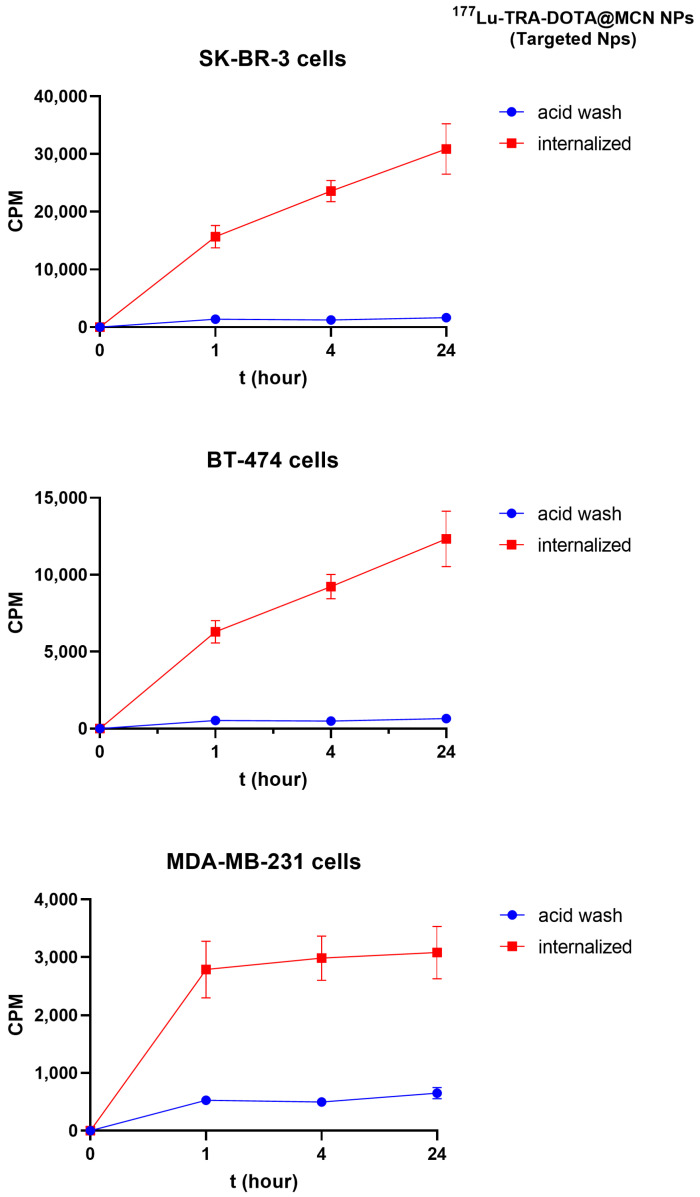
CPM (Radioactivity)/Time Ratios of [^177^Lu]Lu-DOTA@TRA/MC@Si Nanocomposite in SK-BR-3, BT-474, and MDA-MB-231, and Cell Lines. Red curve: “internalized” is defined as the radioactivity of internalized and cell surface-bound fraction; Blue curve: “acid wash” is presented as the radio-activity of the cell surface-bound fraction”. Data are presented as mean values ± SD, determined in triplicate from 2 independent experiments.

**Figure 9:**
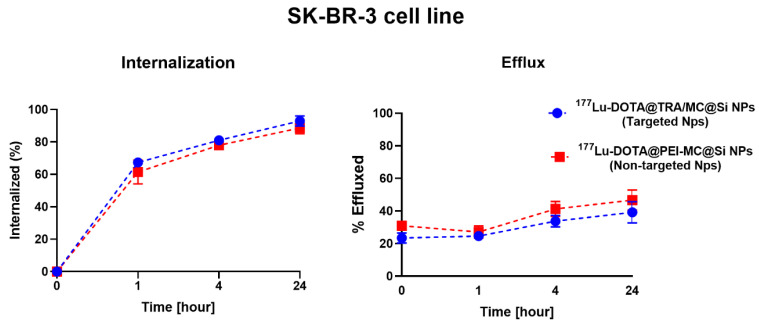
Cellular Internalization and Efflux of [^177^Lu]Lu-DOTA@TRA/MC@Si and [^177^Lu]Lu-DOTA@PEI/MC@Si Nanomaterials in SK-BR-3 Cells. Data are presented as mean values ± SD, determined in triplicate from 2 independent experiments.

The internalization results of [^177^Lu]Lu-DOTA@TRA/MC@Si in MDA-MB-231 (negative control cell line) and SK-BR-3 (target cell line) are illustrated in Figure 8. The intracellular radioactivity level in the SK-BR-3 cell line was 5.6 times higher than that in the MDA-MB-231 cell line during the initial hour of incubation. Figure 9 provides the percentages of internalization and efflux for [^177^Lu]Lu-DOTA@TRA/MC@Si and [^177^Lu]Lu-DOTA@PEI/MC@Si in the SK-BR-3 cell line. According to the data, 68% of the trastuzumab-targeted nanomaterial ([^177^Lu]Lu-DOTA@TRA/MC@Si) was internalized after 1 h, however, the non-trastuzumab-structured nanocomposite [^177^Lu]Lu-DOTA@PEI/MC@Si) had an internalization value of 61%. At 24 h, both nanomaterials exhibited a 90% internalization value. The efflux values were comparable for both nanomaterials, measured at approximately 20% at the first hour and 40% after 24 h.

As it is known that cellular uptake of TRA occurs via clathrin-dependent endocytosis, we were interested in studying the internalization of our ^177^Lu-labeled nanocomposites to confirm the mechanism of cellular retention as an important property of their potential therapeutic efficacy. In our experiments, the enhanced uptake of the [^177^Lu]Lu-DOTA@TRA/MC@Si nanocomposite in the HER2-positive SK-BR-3 cell line was evident when compared to the non-targeted MDA-MB-231 cell line. Specifically, the SK-BR-3 cells demonstrated 5.6-fold higher radioactivity in the initial hour. Furthermore, while both the trastuzumab-targeted and non-targeted nanocomposites reached a 90% internalization value by 24 h, the former showed a marginally superior initial internalization. However, the efflux values for both nanocomposites were equal, suggesting similar intracellular retention over time. These findings suggest that the TRA antibody as a targeting vector did not mediate the cellular uptake of [^177^Lu]Lu-DOTA@TRA/MC@Si. However, the obtained results are consistent with similar studies in the literature, demonstrating that the impact of the antibody on intracellular uptake is a complex process.

The interaction between nanoparticles and cells is crucial for their therapeutic efficacy. This interaction is primarily determined by the physicochemical properties of the nanoparticles, such as size, shape, charge, and surface functionalization [47,48,49,50]. The unique characteristics of nanoparticles, including a small size and a substantial surface area-to-volume ratio, significantly influence cellular processes such as adhesion, uptake, trafficking, and exocytosis. Notably, surface characteristics play a vital role in nanoparticle applications in fields like phototherapy, imaging, and drug/gene delivery, emphasizing the necessity for controlled interactions between nanoparticles and cells. These interactions are significantly influenced by the physicochemical attributes of the nanoparticles and the cellular context rather than merely by the presence of targeted antibodies.

In a 2017 study by Cai et al., trastuzumab was conjugated with gold nanoparticles and labeled with ^177^Lu, creating trastuzumab-AuNP-^177^Lu. This complex demonstrated a binding affinity of 7.6 ± 2.0 nM and bound to about 25% of HER-2 receptors on SK-BR-3 cells. Internalization studies revealed significantly higher uptake of trastuzumab-AuNP-^177^Lu compared to AuNP-^177^Lu, peaking at 5 h with 76 ± 2 uptake, and maintaining higher intracellular retention and activity over time. This indicates enhanced targeting and retention of trastuzumab-AuNP-^177^Lu in HER-2 positive cells [29]. Similarly in a study by Trujillo-Nolasco et al. (2019), uptake studies were conducted on the RAW 264.7 cell line with [^177^Lu]Lu-DOTA-HA-PLGA and [^177^Lu]Lu-DOTA-HA-PLGA-(Methotrexate-MTX) nanomaterials [51]. Evaluation of the study results showed an 8-fold increase in the uptake of nanomaterials when HA is present, as seen with [^177^Lu]Lu-DOTA-HA-PLGA (drug-free NP) and [^177^Lu]Lu-DOTA-HA-PLGA (MTX) (drug-carrying NP). In cells where receptors were previously blocked with HA, the uptake value decreased by ~40% compared to the group with unblocked receptors. Typically, extracellular HA is endocytosed into the cell’s lysosomes. The internalization test showed increasing proportionally with the uptake of nanomaterials, with at least 10% of the associated activity found in the cell’s cytoplasmic compartment. Notably, the internalization behavior in cells with blocked receptors was similar to that in cells with unblocked receptors.

It has been demonstrated that the presence of hyaluronic acid (HA) on the surface of nanoparticles enhances specific interaction with CD44 receptors, thereby facilitating targeted uptake (through [^177^Lu]Lu-DOTA-PLGA (MTX) and other HA-modified formulations). Moreover, the study revealed that even when CD44 receptors are blocked, passive uptake occurs and that physical interactions between nanoparticles and the cell surface, as well as cell membrane permeability, also contribute to uptake. This study’s results emphasize that while targeted uptake is generally more effective, the importance of passive uptake cannot be ignored. It is thought that passive uptake may be dependent on factors such as decreased receptor expression during cell culture, loss of antibodies on the nanoparticles, and inefficient conjugation of ligands. Therefore, contrary to receptor-mediated uptake previously emphasized in the literature, this study concludes that both passive and targeted uptake mechanisms play a role in the internalization of nanoparticles. Future studies should focus on detailed quantitative analyses to reassess the stability and functionality of nanoparticle-ligand conjugation and better distinguish between passive and targeted uptake mechanisms. When comparing the internalization and efflux values of the [^177^Lu]Lu-DOTA@TRA/MC@Si and [^177^Lu]Lu-DOTA@PEI/MC@Si nanomaterials in SK-BR-3 cells obtained in this study, similar inferences can be made. Indeed, the internalization values between the two nanocomposites are quite similar.

## 3. Material and Method

All chemicals used in the synthesis stage of the nanomaterial are of analytical purity, and trastuzumab and DOTA-NHS-ester were obtained from Biozol Diagnostica (Biozol GmbH, Eching, Germany) and Macrocyclics (Dallas, TX, USA) companies, respectively.

### 3.1. Synthesis of Polyethyleneimine Integrated Mesoporous Carbon@Silica (MC@Si) Nanocomposite

As described in our previous paper [43], the initiation of MCNp synthesis began with combining phenol, formalin, and a NaOH solution. This mixture was then heated and stirred upon the addition of a solution containing Pluronik^®^F127 (Sigma-Aldrich, Taufkirchen, Germany). Following the hydrothermal reaction, the resultant particles were separated by centrifugation and lyophilized. The calcination process was applied to obtain graphitic MCNp. Subsequently, the synthesis of the MC@Si nanocomposite was initiated by preparing a solution containing CTAB (hexadecyltrimethylammonium bromide) and calcined MCNp. This solution was homogenized through the application of ultrasonication. NaOH was then added to this mixture, and the solution was heated. After establishing an inert atmosphere with argon gas, tetraethyl orthosilicate (TEOS) and 3-(triethoxysilyl)propionitrile (TESPN) were added, and the mixture was stirred. Upon the separation of the supernatant by centrifugation, the MC@Si nanocomposite was obtained. The surface of the MC@Si nanocomposite was modified to form -COOH groups. This was achieved by refluxing the MC@Si in a sulfuric acid solution. The resultant -COOH-MC@Si was separated from the acid solution and evaluated through FTIR analysis. The surface of -COOH-MC@Si was modified with polyethyleneimine (PEI). This is accomplished by dissolving and combining -COOH-MC@Si and PEI in ethanol. After the application of sonication, the material was washed with PBS and stored at 4 °C for further usage.

According to the results, the size and surface charges of the synthesized nanomaterial were determined using dynamic light scattering (DLS) and Zeta potential measurements. Transmission electron microscopy (TEM) and Fourier transform infrared spectroscopy (FTIR) analyses confirmed that the structural properties of the nanocomposites were preserved and exhibited characteristic bands. The efficient integration of TRA enhanced the targeting capability of the nanocomposite. These results demonstrate that the nanocomposite meets the expected structural and functional properties and holds potential for advanced applications. Further details on the detailed characterization of the nanomaterial can be found in our previous publication [43].

### 3.2. Determination of Primary Amine Groups with the Ninhydrin Method

Prior to DOTA conjugation, the concentration of available free amine groups on the surface of the PEI-MC@Si nanocomposite was determined using the ninhydrin test [45]. For this purpose, 5 mg/500 µL of PEI-MC@Si nanocomposite was pipetted into a microcentrifuge tube, homogenized, and centrifuged. The resulting pellet was then resuspended in 200 µL of 60% ethanol, and each solution of the nanocomposite was transferred to glass test tubes. Subsequently, 100 µL of potassium cyanide (KCN) and 75 µL of phenol, both components of the Kaiser kit, were sequentially added to the tubes. After vortexing for 10 s, 75 µL of ninhydrin reagent was added to each tube. The tubes were sealed with glass beads and heated in a 97 °C oil bath for 10 min, then cooled in ice water. The cooled nanosolutions were transferred to 15 mL Falcon tubes and diluted to 5 mL with 60% ethanol. After centrifugation at 10,000 rpm for 20 min, the supernatant was transferred to new tubes, with an additional 200 µL of solution pipetted from each. These volumes were then adjusted to 1 mL with 60% ethanol. From these solutions, 200 µL samples were taken in triplicate and added to 96-well plates, and their absorbance at 570 nm was measured using a microplate reader. Concurrently, a calibration curve was established using octylamine at a minimum of five concentrations, following the same procedure. The concentration of amine groups on the PEI-MC@Si nanocomposite was then determined using this calibration curve [45].

### 3.3. Conjugation of DOTA-NHS-Ester to the Nanocomposite

The conjugation with DOTA-NHS-ester was performed using previously described procedures [46,52,53]. Initially, the DOTA-NHS-ester was dissolved in dimethyl sulfoxide (DMSO) at a concentration of 10 mg/mL to prepare the main stock solution. Since the amine concentration in the PEI-MC@Si nanocomposite was determined by the ninhydrin test, different concentrations of DOTA-NHS-ester, corresponding to 1 mg of the nanocomposite, were selected. Subsequently, 10 µL of DOTA-NHS-ester from the main stock was pipetted and diluted in 10 mL of 0.25 M ammonium acetate buffer solution (pH 8.3). Thus, the obtained DOTA-NHS-ester concentrations were prepared to correspond to 50, 100, and 200 times the molar excess of the amine concentration on the surface of the PEI-MC@Si nanocomposite. Additionally, a control group was prepared using an equal volume of DMSO only. The reactions were incubated at a constant rotation of 500 rpm at 23 °C for 24 h and were stopped by adding a 10% (*v*/*v*) 1.5 M Tris–HCl buffer solution (pH 8.3). Then, the solution of the DOTA-linked nanocomposite was centrifuged at 10,000 rpm for 30 min at 4 °C, and the upper phase was separated for the detection of the loaded DOTA chelator concentration. Finally, the pellet was washed three times with PBS and stored at 4 °C for subsequent uses. The purity of the nanocomposites was confirmed by the precise calibration of amine concentrations using the ninhydrin method, ensuring an accurate assessment of surface functionalization.

### 3.4. Determination of the DOTA Content in Nanocomposites by the Arsenazo(III) Method

The concentration of DOTA in nanocomposites was determined by the Arsenazo(III) method as described in the study by Al-Ejeh et al. [46]. To reduce the volume of the solution used and standardize the method, 96-well plates were utilized. Initially, a Cu:Arsenazo(III) stock solution was prepared. For this purpose, 100 µL of 1 mg/mL standard Cu atomic absorption solution, 0.776 mg of Arsenazo(III), and 3 mL of metal-free 5 M ammonium acetate (NH_4_OAc) buffer were combined in a 15 mL falcon, and the final volume was adjusted to 10 mL with ultrapure water. The prepared dark blue solution was preferably used immediately before each experiment but could be stored in the dark at room temperature for subsequent applications. Standard DOTA concentrations were prepared using dissolved DOTA-NHS-ester in ultrapure water. 10 µL were allocated into the wells from both the upper phases of the DOTA@PEI-MC@Si nanocomposite, where DOTA concentrations were to be identified and from the standard DOTA solutions with known concentrations. Then, 190 µL of the previously prepared stock Cu:Arsenazo(III) solution was added to these wells. Finally, the 96-well plates were incubated at 37 °C for 30 min, and after the completion of the incubation period, the absorbance of each well was measured at 595 nm. The relationship between the absorbance of the Cu:Arsenazo(III) reagent and the concentration of the DOTA standard solutions and DOTA@PEI-MC@Si nanocomposites was revealed by the interpolation of the standard curve graph created. The amount of DOTA per mass of nanocomposite was calculated to be 90.2 ± 2.8% of the initially added DOTA-NHS-ester, indicating a high level of successful reaction with the nanocomposite’s amine groups.

### 3.5. Attachment of Trastuzumab to the Structure of DOTA@PEI-MC@Si Nanocomposite

The integration of trastuzumab (TRA) into the DOTA@PEI-MC@Si nanocomposite was carried out by the amidation method. According to this method, the pH of the MES buffer (0.1 M, pH 4.7) and PBS (0.1 M, pH 7.4) used in this reaction is crucial. Before the synthesis, both buffer systems were freshly prepared at the desired pH and warmed to room temperature. According to the procedure, 125 µL of trastuzumab (1 mg/mL) was dissolved in PBS. Then, 40 mg of N-ethyl-N′-(3-dimethylaminopropyl) carbodiimide hydrochloride (EDC) and 10 mg of NHS were dissolved in 2 mL of MES buffer and immediately added to the trastuzumab solution. The solution was stirred at high rpm at room temperature for 30 min under a magnetic stirrer. Then, 1 mg of the DOTA@PEI-MC@Si nanocomposite was dissolved in 2 mL of PBS added to the mixture, and stirred for 4 h [54]. At the end of the reaction, to remove the excess trastuzumab that did not conjugate into the nanocomposite structure, it was centrifuged at 10,000 rpm for 15 min with PBS, and this process was applied twice. Then, the pellet was stored at −20 °C to be used for radiolabeling experiments, and the supernatant was applied to the BCA test to determine the concentration of TRA conjugated to the nanocomposite structure. To perform the test, BSA standard solutions were prepared at seven different concentrations and pipetted and added to 96-well microplates. In the experiment, a total of 200 µL was added to the 96-well microplate wells, 100 µL of which was the sample to be tested and 100 µL was the BCA solution. To prepare the BCA solution, a total of 1.5 mL of A solution, 1.5 mL of B solution, and 60 µL of C solution was added to each well, in multiples of 100 µL of A solution, 100 µL of B solution, and 4 µL of C solution. A total of 3.06 mL of solution was prepared, and this solution was added to the 100 µL samples taken from the supernatant after the synthesis stage of DOTA@TRA/MC@Si nanocomposite was completed. The analytical data indicate that the binding efficiency of trastuzumab to the DOTA@PEI-MC@Si nanocomposite was 31.1 ± 4.5%, demonstrating a significant incorporation of the antibody into the nanocomposite structure.

### 3.6. Radiolabeling Studies of the Nanocomposite with ^177^Lu

For the optimization studies, 1 mg/mL DOTA@TRA/MC@Si nanocomposite was first dispersed in ultrapure water and homogenized for 2–3 min. To evaluate the impact of material quantity on the RCY, the nanocomposite was prepared at varying concentrations (50, 100, 250, 500 µg/µL). The volume of each concentration was adjusted to 500 µL with 0.5 M pH 5 HEPES solution, while all other experimental conditions were kept constant. Simultaneously, the effects of differing incubation times (1, 3, 6, 24 h), temperatures (24, 37, 42 °C), and pH values (3, 3.5, 4, 5, 7) on the RCY were investigated. Parallel radiolabeling studies were conducted with the non-targeted version of the nanoparticle, DOTA@PEI-MC@Si, under identical conditions.

The radiolabeling of the DOTA@TRA/MC@Si nanocomposite with ^177^Lu was performed under the optimized conditions determined from these studies. The nanocomposite was again homogenized in metal-free ultrapure water at a concentration of 1 mg/mL. For the chelation process, 200 µL of 0.5 M HEPES solution was mixed with the 100 µg/µL DOTA@TRA/MC@Si nanocomposite solution, followed by sonication for about 30 min. Subsequently, 37 MBq [^177^Lu]LuCl_3_ in 2.5 µL of 0.01 M HCl was added, ensuring the pH was stabilized at 4.5. The mixture was then sonicated for approximately an hour to facilitate the radiolabeling reaction and left to incubate overnight in a thermomixer set at 42 °C.

Post-radiolabeling, to extract unreacted ^177^Lu and enhance the purity, the nanocomposite was subjected to centrifugation at 10,000 rpm for 30 min using a 30 kDa MWCO filter. The supernatant was discarded, and the pellet was rinsed with PBS (0.5 M pH 8) and resuspended in 0.1 M pH 7.4 PBS. The RCY and purity were determined using the thin layer radio chromatography (TLRC) method. For this, 1 µL of the [^177^Lu]Lu-DOTA@TRA/MC@Si solution was applied onto an instant thin layer chromatography strip (iTLC-SG) that was placed in a chamber with the prepared mobile phases of 0.1 M sodium citrate (pH 5)/water (1:5) and 10 mM DTPA. Once the mobile phase ascended to the top, the iTLC-SG strip was dried, and the radiolabeled spots were analyzed using a phosphor imager (CR-35 Bio, Elysia-Raytest, Straubenhardt, Germany), utilizing the Aida Image Analysis software (version 4.21, Elysia-Raytest, Straubenhardt, Germany) for evaluation. Each ITLC-SG strip was exposed to the imaging screen for a duration of 2 min to ensure accurate analysis [29,32].

### 3.7. Determination of Stability in Serum and Lipophilicity of [^177^Lu]Lu-DOTA@TRA/MC@Si Nanocomposite

The determination of stability was performed in accordance with a previously described thin layer radiochromatography (TLRC) method [55]. The stability of the [^177^Lu]Lu-DOTA@TRA/MC@Si nanocomposite was assessed using the TLRC method with iTLC-SG strips on samples taken at different time intervals (0, 1, 24, 48, 72 h) using 0.1 M sodium citrate/water (1:5) with 10 mM DTPA as the solvent. In the experiment, 4.76 µL of the [^177^Lu]Lu-DOTA@TRA/MC@Si nanocomposite (100 kBq) was added to each microcentrifuge tube containing 400 µL of human serum and two tubes each containing 400 µL of PBS. These tubes were then incubated at 37 °C with stirring at 500 rpm, with one of the PBS solutions also incubated at room temperature. Samples of 1 µL were taken from each tube at the above-defined time intervals and applied to iTLC-SG strips for analysis by TLRC. The experiments were conducted in triplicate.

The lipophilicity of the [^177^Lu]Lu-DOTA@TRA/MC@Si radioligand was determined by the logD_7.4_ distribution coefficient. A total of 500 µL of PBS (pH 7.4) and n-octanol buffer solutions were initially transferred to three different microcentrifuge tubes. After adding 100 kBq of activity from the [^177^Lu]Lu-DOTA@TRA/MC@Si radioligand to each tube, they were stirred at room temperature for 30 min. Following this period, each tube was centrifuged at 800× *g* for 15 min, and three 100 µL samples were taken from both phases, with their radioactivities measured using a gamma counter (Wallac Wizard, PerkinElmer, Waltham, MA, USA). The count per minute (CPM) activity obtained was calculated as the logarithm of the octanol/PBS ratio, with logD_7.4_ values presented as average values ± standard deviation. Two independent experiments were conducted, each in triplicate.

### 3.8. Cellular Uptake Studies of [^177^Lu]Lu-DOTA@TRA/MC@Si Nanocomposite

For the in vitro studies on cellular uptake, the HER2-positive SK-BR-3 and BT-474 cell lines were utilized. The cells were cultured in McCoy’s 5A and RPMI cell media supplemented with 10% fetal bovine serum (FBS), 1% L-glutamine, 1% non-essential amino acids (NEAA), and 1% sodium pyruvate, and maintained at 37 °C in a 5% CO_2_ atmosphere. The cells were counted using an automatic cell counter (EVE, NanoEnTek, Waltham, MA, USA) upon reaching confluency, then seeded in a 48-well plate at a density of 1 × 10^5^ cells per well and incubated for 48 h. The HER2-negative MDA-MB-231 cell line was also included in the experiment as a control group, with cells seeded and incubated similarly to the SK-BR-3 and BT-474 cells. After the incubation period, each well was washed once with 300 µL of cell culture medium. Then, 50 µL of the [^177^Lu]Lu-DOTA@TRA/MC@Si solution was added to 450 µL of cell medium in each well, resulting in a total activity of 0.1 MBq per well (*n* = 3). The plates were then incubated at 37 °C for 1, 24, 48, 72, and 96 h. Following each incubation period, the plates were placed on a tray containing ice. The cells were washed twice with 300 µL of cold PBS and then treated with 400 µL of warm 0.1 M NaOH solution (37 °C) for 5–7 min, leading to cell disintegration. The cell solution from each well was then transferred to Wallac tubes, and the radioactivity were measured using a gamma counter (Wallac Wizard, PerkinElmer, Waltham, MA, USA; *n* = 3).

Subsequently, the uptake of [^177^Lu]Lu-DOTA@TRA/MC@Si corresponding to 1 mg of protein in each tube was determined using the Bradford method [55]. In this assay, 10 µL samples were taken from the Wallac tubes after the [^177^Lu]Lu-DOTA@TRA/MC@Si radioactivity measurements were completed and transferred to a 96-well plate. For the control group, 10 µL of NaOH was transferred to the bottom row of the plate. Additionally, 10 µL of each different concentration of protein standard was transferred to the 96-well plates. Then, 90 µL of Bradford reagent was added to each well and the plate was left at room temperature for 7 min. Finally, the plates were read at 595 nm using a microplate reader, and the absorbance values corresponding to the protein concentration in the wells were determined.

### 3.9. Competitive Binding Experiments of Radiolabeled Nanocomposite

Incubation and measurements were conducted in accordance with a previously described method [55] along with a few modifications. SK-BR-3 and BT-474 cells were counted using an automatic cell counter (EVE, Nano EnTek, Waltham, MA, USA) and seeded into a 48-well plate at a density of 1 × 10^5^ cells per well and incubated for 48 h. After the incubation period, the supernatant was removed from each well, and the cells were washed once with 100 µL of culture medium. Subsequently, trastuzumab suspended in a culture medium at various concentrations (1 nM–5000 nM) was added to each well in a volume of 90 µL. Immediately afterward, 10 µL of the [^177^Lu]Lu-DOTA@TRA/MC@Si radiolabeled compound (100 kBq) was added to the wells, and the plates were incubated at 37 °C for 24 h. After incubation, the plates with seeded cells were placed on ice, and the cell medium in the wells was removed. The wells were then washed once with 200 µL of culture medium and once with 200 µL of cold PBS. Each well was treated with 200 µL of warm 0.1 N NaOH (37 °C) for 5–7 min. The cell content from each well was then transferred to Wallac tubes, and the radioactivity in each tube was measured with a gamma counter (Wallac-Wizard, PerkinElmer, Waltham, MA, USA) The experiments were conducted twice with *n* = 4 replicates.

### 3.10. In Vitro Cellular Uptake (Internalization) and Efflux Studies

The cell culture and seeding of cells was as described under Section 3.8. Internalization and efflux studies were conducted by a previously described method [55]. After the incubation period, each well was washed once with 300 µL of cell culture medium. The MDA-MB-231 cell line was also included in the experiment set as a negative control group for the internalization study, with cells seeded into different plates for each time point (1, 2, 4, and 24 h).

#### 3.10.1. Internalization

After washing the cells, 450 µL of cell medium was added and 50 µL of [^177^Lu]Lu-DOTA@TRA/MC@Si (targeted Np) or [^177^Lu]Lu-DOTA@PEI/MC@Si (non-targeted Np) radiolabeled compounds was added to the wells, with a total activity of 0.1 MBq per well (*n* = 3). The plates were incubated at 37 °C for 1, 2, 4, and 24 h. After the incubation periods, each plate was placed in a tray containing ice, and the cells were washed twice with 300 µL of cold PBS. Then, they were washed for 2 min with 500 µL of cold acidic wash solution (0.02 M NaOAc, pH 5). The wash solutions were transferred to Wallac tubes for counting. This process was repeated twice for each well. After this process, each well was treated with 500 µL of warm 0.1 M NaOH solution (37 °C) for 5–7 min to disintegrate the cells. The disintegration of the cells was verified under a microscope. Then, the cell solution of each well was transferred to Wallac tubes and the radioactivity of each tube was measured using a gamma counter (Wallac Wizard, PerkinElmer, Waltham, MA, USA; *n* = 3). The experiments were conducted twice.

#### 3.10.2. Efflux

After completing the cell seeding under the conditions above-mentioned, 50 µL of the [^177^Lu]Lu-DOTA@TRA/MC@Si (targeted Np) and [^177^Lu]Lu-DOTA@PEI/MC@Si (non-targeted Np) radiolabeled compounds were added separately to the wells, with a total activity of 0.1 MBq per well (*n* = 3), in addition to 450 µL of cell medium. The plates were then incubated at 37 °C for 24 h. After the incubation period, each plate was placed in a tray containing ice, and the cells were washed once with 300 µL of cold PBS. Then, each well was treated with 500 µL of cold acidic wash solution (0.02 M NaOAc, pH 5) and left for 2 min before being washed twice. After a final wash with 500 µL of PBS solution, 500 µL of fresh culture medium (37 °C) was added to each well, and incubation was carried out at 37 °C for 0 (immediately after adding the culture medium), 1, 2, 4, and 24 h. After the incubation periods, the culture medium in the wells was removed and transferred to Wallac tubes for radioactive counting. Each well was then washed once with cold PBS, and after removing the cells from the surface they were adhered to with 500 µL of 0.1 M NaOH, they were again transferred to Wallac tubes for counting. Thus, efflux values were calculated as a percentage by dividing the radioactive count of the supernatant by the total radioactive count of the supernatant and lysed cell solution. The experiments were conducted twice with *n* = 4 replicates.

## 4. Conclusions

In this study, the TRA/PEI-MC@Si nanocomposite was labeled with ^177^Lu to evaluate its potential for radiotherapy. Various parameters influencing RCY were examined and optimum conditions were identified. The in vitro cellular uptake of radiolabeled nanocomposites was assessed in the SK-BR-3, BT-474, and MDA-MB-231 cell lines. The [^177^Lu]Lu-DOTA@TRA/MC@Si nanocomposite, due to its structural incorporation of trastuzumab, consistently showed higher uptake values in every time interval when compared to nanocomposites that were not TRA-conjugated. [^177^Lu]Lu-DOTA@PEI-MC@Si and [^177^Lu]Lu-DOTA@TRA/MC@Si radiolabeled nanocomposites displayed lower uptake values in the HER2-negative MDA-MB-231 cell line. These findings suggest that the [^177^Lu]Lu-DOTA@TRA/MC@Si nanocomposite has a higher affinity for cells carrying the HER-2 receptor. It was observed that the examined nanocomposites were moderately lipophilic and that the radiochemical yield varied depending on parameters such as the amount of nanocomposite, incubation time, temperature, and pH. The optimum radiolabeling conditions were determined to be the incubation of the nanocomposite at a concentration of 100 µg/µL for 24 h at a pH of 4.5 and a temperature of 42 °C. In vitro, the [^177^Lu]Lu-DOTA@TRA/MC@Si nanocomposite demonstrated cellular uptake in HER2-positive cells, TRA affinity, and cellular internalization capability, thereby suggesting their ability to specifically target HER-2-positive breast cancer cells. However, compared to [^177^Lu]Lu-DOTA@TRA/MC@Si, [^177^Lu]Lu-DOTA@PEI/MC@Si nanocomposites, which were not TRA-conjugated, showed significant passive transport into the. After careful optimization of TRA-dependent cellular uptake of the nanocomposites, the initiation of in vivo studies with the [^177^Lu]Lu-DOTA@TRA/MC@Si nanocomposite in animal models of breast cancer is still needed to further assess their potential as targeted therapeutics for the treatment of HER-2-positive breast cancer.

## Figures and Tables

**Table 1:** Stability of the [^177^Lu]Lu-DOTA@TRA/MC@Si nanocomposite after incubation in 10% HSA and PBS. Data represent the mean values plus minus standard deviation from two independent experiments, each performed in triplicate.

Incubation Time (h)	Radiochemical Purity (%)		
	HSA (37 °C)	PBS (37 °C)	PBS (24 °C)
0	94 ± 0.4	97 ± 0.7	95 ± 0.2
1	74 ± 0.6	96 ± 1.9	90 ± 0.8
24	75 ± 0.6	91 ± 0.4	75 ± 0.3
48	67.2 ± 0.5	72.5 ± 1.2	51.8 ± 0.5
72	62.4 ± 0.7	70.1 ± 0.3	44.2 ± 2.2

**Table 2:** Stability test results of the [^177^Lu]Lu-DOTA@PEI-MC@Si radiolabeled nanocomposite. Data represent mean values plus minus standard deviation from two independent experiments, each performed in triplicate.

Incubation Time (h)	Radiochemical Purity (%)		
	HSA (37 °C)	PBS (37 °C)	PBS (24 °C)
0	93 ± 0.5	96 ± 0.8	93 ± 0.4
1	76 ± 0.6	94 ± 0.9	89 ± 0.5
24	74 ± 0.9	89 ± 0.4	72 ± 0.3
48	64 ± 0.3	71 ± 0.7	55 ± 0.6
72	63 ± 0.7	70 ± 0.4	55 ± 0.2

## Data Availability

The authors confirm that the data supporting the findings of this study are available within the article.
